# Insights into adaptive evolution in different locomotor modes and phylogeny of Sciuridae from mitogenomes of flying and tree squirrels

**DOI:** 10.1371/journal.pone.0355043

**Published:** 2026-08-14

**Authors:** Di Zhao, Zhongsong Wang, Wenyu Song, Wenge Dong

**Affiliations:** Key Laboratory of Ectoparasite Systematics and Evolution of Yunnan Provincial Education Department, Institute of Pathogens and Vectors, Dali University, Dali, China; Instituto Leonidas e Maria Deane / Fundacao Oswaldo Cruz, BRAZIL

## Abstract

Mitogenomes offer valuable insights into evolutionary adaptation. Squirrels have evolved two distinct locomotor modes, gliding and scansorial climbing. To explore whether such locomotor disparities are reflected at the mitogenome level, we newly sequenced the *Priapomys leonardi* mitogenome and compared it with published mitogenomes of gliding (flying squirrels) and scansorial (tree squirrels). Our result revealed highly conserved mitogenomic organization across both tribes, with consistent gene arrangement, nucleotide composition, codon site base composition, and codon usage patterns. Evolutionary rates showed higher Ka/Ks ratios of *ATP8, COX1*, *CYTB*, and *ND5* genes in the Pteromyini than in the Sciurini. Codon usage bias was more than in flying squirrels than in tree squirrels. These results suggested that divergent natural selection acted on these both lineages. We reconstructed the phylogenetic tree of the family Sciuridae. Phylogenetic analysis robustly revealed four monophyletic subfamilies: Ratufinae, Sciurinae, Xerinae, and Callosciurinae. The phylogenetic relationship among these subfamilies was ((Callosciurinae + Xerinae) + Sciurinae) + Ratufinae, with high nodal support. Phylogenetic reconstruction placed *Priapomys leonardi* within Sciuridae, and supported a sister relationship between Pteromyini (flying squirrels) and Sciurini (tree squirrels). This study provides insights into locomotor mode-linked adaptive evolution and offers a genetic foundation for future research on sciurid phylogeny and species evolution.

## 1. Introduction

The flying squirrels (Sciurinae, Pteromyini) are rodent lineage possessing a unique gliding ability via a patagium (gliding membrane) between their forelimbs and hindlimbs. Historically, their taxonomic status has been contentious. Morphological studies have supported their elevation to a higher taxonomic rank. For instance, based on penis morphology, Pocock (1923) concluded that flying squirrels and squirrels should be placed in separate families [1]. Similarly, analyses of dental and crania fossils from the Oligocene by Bruijn and Unay (1989) led them to advocate for separate familial status [2]. Ellerman and Morrison-Scott (1951) categorized the taxa with glide membrane at the tribal level (Pteromyini) [3]. Other morphological studies by Hoffmann et al. (1993) and Wang Yingxiang (2003) also supported separating flying squirrels and squirrels should be classified into distinct familial-level groups based on morphology [4,5]. Wei Fuwen et al. (2021) similarly supported this view, classifing flying squirrels as a separate family [6]. Most early taxonomic revisions were primarily based on morphological comparisons. With the advent of molecular phylogenetic techniques, perspectives on flying squirrels classification have shifted. Studies based on genetic data, such as those by Mercer and Roth (2003) and Steppan et al. (2004), and others divided the family Sciuridae into five subfamilies (Sciurillinae, Ratufinae, Callosciurinae, Sciurinae, and Xerinae) [7,8]. This five-subfamily classification, which nested flying squirrels (Pteromyini) within the subfamily Sciurinae, was now widely adopted on major taxonomic references. These included *Handbook of the mammals of the world* [9], *A Guide to the Mammals of China* [10], and *Catalogue of mammals in China* (2024) [11].

The subfamily Sciurinae comprises two distinct tribes: the gliding Pteromyini and the scansorial Sciurini. Tree squirrels, which belong to the tribe Sciurini (subfamily Sciurinae), exhibit scansorial locomotion specialized for climbing and jumping. Adapted for an agile arboreal life, they utilize sharp claws and hind limbs to climb, leap, and maintain balance among branches. Pteromyini are unique within Sciuridae for their capacity for gliding [9]. To explore whether such locomotor disparities are reflected at the mitogenome level, we compared complete mitogenomes from 9 species in Pteromyini and 24 species in Sciurini.

In this study, we newly sequenced the *Priapomys leonardi* (Thomas 1921) mitogenome for the first time and performed comparative analysis of mitogenomes between the gliding Pteromyini and the scansorial Sciurini, integrating publicly available data from NCBI. Our study aimed to investigate the possible consequences of divergent locomotory modes on mitogenome. Additionally, we reconstructed phylogenetic trees to explore the evolutionary relationships within Sciuridae.

## 2. Materials and methods

### 2.1. Specimen collection and DNA extraction

An adult specimen of *Priapomys leonardi* was collected at an elevation of 3000 m in Weixi Lisu Autonomous County, Yunnan. The captured *Priapomys leonardi* received isoflurane inhalation anesthesia. A dual euthanasia method combining isoflurane overdose and cervical dislocation was applied for humane euthanasia. To alleviate pain and distress, we minimized confinement duration during field operation. Animals were kept in shaded temporary cages with supplementary food prior to experimental manipulation. The specimen was preserved at the Institute of Pathogens and Vectors of Dali University (Dali, China). Muscle tissue from this specimen was stored at −80°C for subsequent DNA extraction. For this procedure, tissue preserved in 95% ethanol was first from EP tube, rinsed in sterile distilled water for 30 minutes, and then minced tissue using sterile surgical scissors. Genomic DNA was extracted from the minced tissue using the DNeasy Blood and Tissue Kit (QIAGEN, Redwood City, CA, USA). The extracted DNA was randomly sheared into 300−500 bp fragments, and was used to construct an Illumina PE library. The mitogenome was subsequently sequenced on Illumina Novoseq 6000 platform (Winnerbio, Shanghai, China). All animal procedures were approved by the Animal Ethics Committee of Dali University (Approval No. 2021-P2-162). Field collection and animal handling complied with institutional and national animal welfare guidelines. No additional field site access permits were required for this study, as all sampling was carried out on non-protected, privately owned land with prior landowner consent.

### 2.2. Assembly, annotation, and analysis of mitogenome sequences

Geneious Prime11.1.5 [12] was used to verify the assembly accuracy of assembly results. Contigs were de novo assembled from Illumina sequence reads using Geneious, with mapping based on relatively conserved *COX1* and *rrnS* sequences. Assembly parameters were set to a minimum overlap 150 bp and minimum overlap identity 99 ~ 100%. Protein-coding genes and rRNA genes were identified by BLAST2.16 [13] and MITOS2WebServer [14], tRNA genes were predicted with tRNAscan-SE 2.0 [15], and ARWEN 1.2 [16], followed by manually checking and correction. The annotated *P. leonardi* mitogenomic sequence has been deposited in GenBank (accession number: PQ468466). Nucleotide composition and codon usage bias were analyzed using cowdonW. Strand asymmetry was calculated as AT-skew = (A − T)/(A + T) and GC-skew = (G − C)/(G + C). DnaSP v6 [17] and MEGA 11 [18] were used to jointly analyze evolution rate of 13 protein-coding genes (Nonsynonymous substitution rate (Ka), synonymous substitution rate (Ks)). The ratio of non-synonymous to synonymous substitutions (Ka/Ks) is a key indicator of selective pressure: Ka/Ks > 1 indicates positive selection, Ka/Ks = 1 indicates neutral selection, and Ka/Ks < 1 indicates purifying selection [19,20]. Further codon usage analysis, including Parity rule 2 (PR2), ENC-Plot (Effective Number of Codon, ENC), Corresponding analysis (COA) and relative synonymous codon usage (RSCU) were performed using RStudio v4.3.1. The circular mitogenome map was drawn with OGDRAWv1.3.1 [21].

### 2.3. Phylogenetic analysis

To infer the phylogeny within Sciuridae, we constructed phylogenetic trees based on two datasets (PCG matrix (protein-coding gene sequences) and PCGRNA matrix (protein-coding genes and rRNAs gene sequences)). *Glis glis* (Linnaeus, 1766) and *Castor fiber*, Linnaeus, 1758 were selected as outgroups (see S1 Table for species details). All phylogenetic analyses were performed using PhyloSuite v2 [22,23]. Multi-sequence alignment was performed using MAFFT v7.313 [24] and subsequently optimized for coding sequences using MACSE v2.0 [25]. To improve alignment quality, poorly aligned positions and gaps were removed with Gblocks 0.19b [26] for protein-coding genes and rRNA sequences, and trimAI v1.4.rev15 [27] for other sequences. Maximum likelihood (ML) analysis was conducted with IQ-TREE v2.2.2.7 [28]. The best-fit substitution model (GTR + F + R4) was selected using ModelFinder v2.2.0 [29] as implemented in IQ- TREE v2.2.2.7. Branch support was assessed with 1000 ultrafast bootstrap replicates, reported as bootstrap support (BS) values. Bayesian inference (BI) analysis was performed using MrBayes v3.2.6 [30] under the GTR + I + G model (also selected by ModelFinder v2.2.0). Two independent runs were executed, each with four independent Markov Chain Monte Carlo (MCMC) chains for 100,000 generations, sampling trees every 1000 generations. The first 25% of sampled trees were discarded as burn-in, and a consensus tree was generated from the remaining samples. Nodal support was presented as posterior probability (PP). The phylogenetic tree was visualized and annotated using iTOL v7 [31] and further edited using Adobe Illustrator 2021 for presentation.

## 3. Results

### 3.1. Comparison of morphological characteristic and mitogenomic organization between Pteromyini and Sciurini

**Fig 1:**
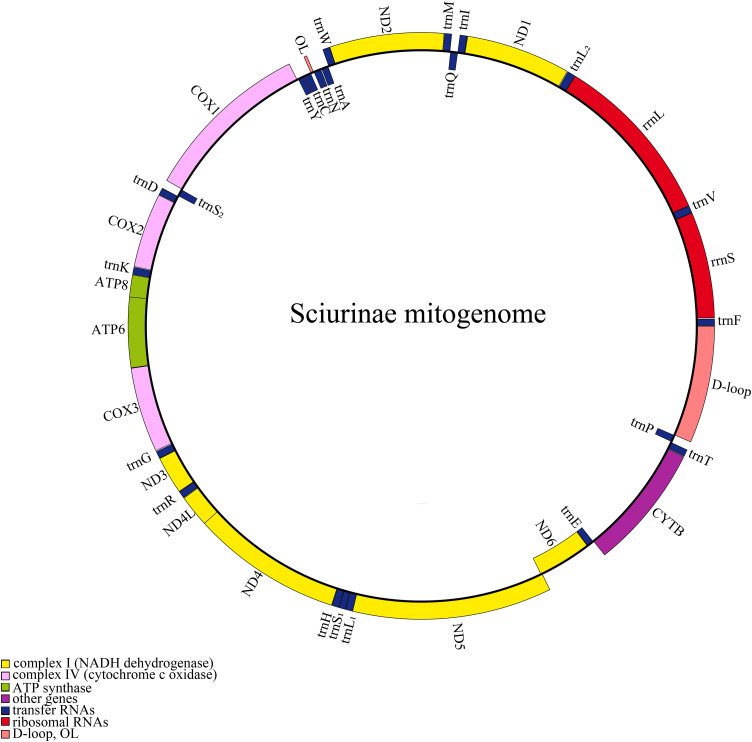
The circular map of the Sciurinae mitogenome.

Pteromyini and Sciurini share close taxonomic and similar dentition, yet differ markedly in locomotor modes, circadian rhythms, and body morphology. Pteromyini are nocturnal gliders and possess a patagium, and exhibit substantially greater variation in body length, tail length, hind foot length, and ear height compared to Sciurini (Table 1). In contrast, Sciurini show a relatively concentrated range of body and tail sizes, resulting in a comparatively uniform overall morphology (Table 1). Mitogenome size of Pteromyini ranged from 16,502–16,620 bp in Pteromyini and from 16,504–16,524 bp in Sciurini (S1 Table). Gene arrangement was identical in both tribes: the mitogenome is a double-stranded, closed circular DNA molecule containing 13 protein-coding genes, 2 rRNA genes, 22 tRNA genes, one non-coding control region (D-loop) region and one origin of light-strand replication (OL) region (Fig 1). With the exception of *ND6* and 8 tRNA genes (*trnQ*, *trnA*, *trnN*, *trnC*, *trnY*, *trnS*_*2*_, *trnE* and *trnP*) which were encoded on the light strand (L-strand), all other genes are encoded on the heavy strand (H-strand).

**Table 1:** Morphological comparison between Pteromyini and Sciurini.

	Pteromyini	Sciurini
Gliding membrane	Absence	Presence
Locomotor Mode	Gliding	Scansorial
Circadian rhythm	Nocturnal	Diurnal
Body size	Large-, medium-, and small-sized	Large-, medium-, and small-sized
Dental Formula	1.0.2.3/1.0.1.3 = 22	1.0.2.3/1.0.1.3 = 22
Head-body length (mm)	117-610	112-370
Tail length (mm)	80-615	82-330
Hindfoot length (mm)	21-90	28-82
Ear length (mm)	13-59	8-43

Morphological data is sourced from [5,9–11].

### 3.2. Comparison of mitogenomic nucleootide composition between Pteromyini and Sciurini

**Fig 2:**
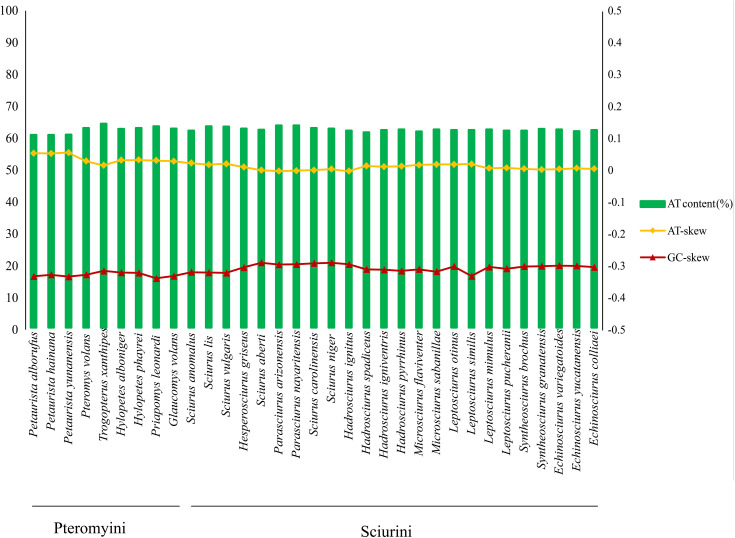
Nucleotide composition analysis of Pteromyini and Sciurini.

**Fig 3:**
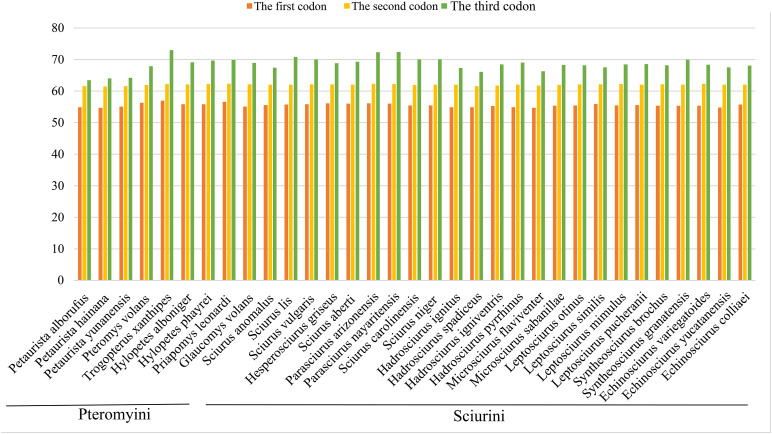
Comparative base composition at codon sites between Pteromyini and Sciurini.

We analyzed the nucleotide composition, including AT content, AT-skew, and GC-skew across the mitogenomes of nine species in Pteromyini and 24 species in Sciurini (Fig 2). The overall AT content ranged from 60.4% to 63.9% in Pteromyini, and from 61.2% to 63.4% in Sciurini. In Pteromyini, AT-skew was consistently positive (0.015–0.055), while GC-skew was negative (−0.339 to −0.316). Among Sciurini, AT-skew was predominantly positive, ranging from −0.003 to 0.022, with negative values observed only in *Parasciurus arizonensis* and *Hadrosciurus ignitus*. GC-skew was uniformly negative (−0.332 to −0.29) in Sciurini. Analysis of base composition at codon positions showed that the third codon position showed the highest AT content in both tribes, exceeding that of the first and second positions (Fig 3).

### 3.3. Comparison of evolutionary rates between Pteromyini and Sciurini

**Fig 4:**
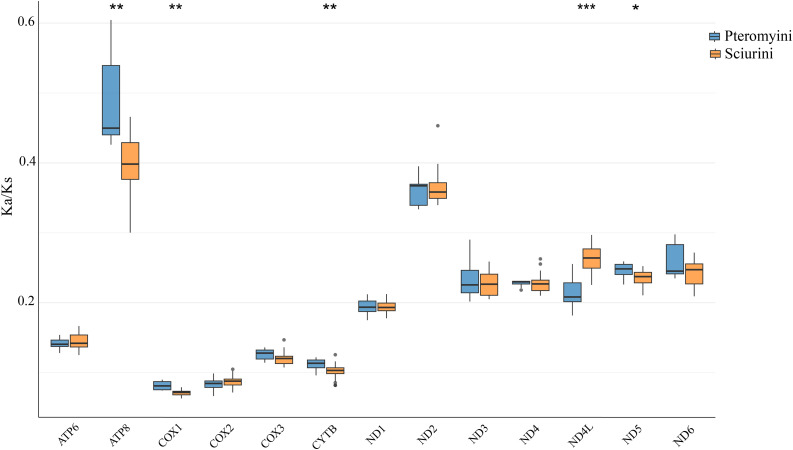
Comparison of evolutionary mutation rates (Ka/Ks) for protein coding genes between Pteromyini and Sciurini. The asterisk (*) denotes statistical significance: *P < 0.05, **P < 0.01, ***P < 0.001.

The average Ka/Ks ratio for each protein-coding gene was calculated in both Pteromyini and Sciurini. Overall, gene ranking based on Ka/Ks ratios were similar in both tribes, with *ATP8* gene consistently showing the highest Ka/Ks ratio and *COX1* had the lowest. The complete ranking for Pteromyini (from highest to lowest) was *ATP8* > *ND2* > *ND6* > *ND5* > *ND4* > *ND3* > *ND4L* > *ND1* > *ATP6* > *COX3* > *CYTB* > *COX2* > *COX1*, For Sciurini, the ranking was: *ATP8* > *ND2* > *ND4L* > *ND6* > *ND5* > *ND3* > *ND4* > *ND1* > *ATP6* > *COX3* > *CYTB* > *COX2* > *COX1*. In both tribes, all 13 PCGs exhibited Ka, Ks, and Ka/Ks ratios less than 1 (S2 Table). Comparative analysis revealed differences in the average Ka/Ks ratios for five genes: *ATP8, COX1*, *CYTB*, *ND4L*, and *ND5*. Specifically, Pteromyini had higher ratios for *ATP8, COX1*, *CYTB*, and *ND5*, whereas Sciurini showed a higher average Ka/Ks ratio for *ND4L*. The remaining genes showed no differences in both tribes. (Fig 4).

### 3.4. Comparison of relative synonymous codon usage and codon usage bias between Pteromyini and Sciurini

**Fig 5:**
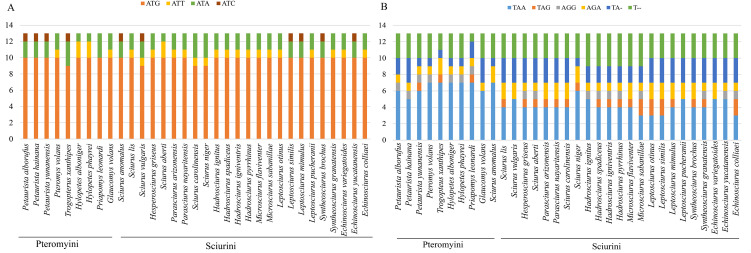
Usage of start codons (A) and stop codons (B) in Pteromyini and Sciurini.

Protein-coding genes in Pteromyini and Sciurini showed similar patterns for start and stop codons. ATG was the predominant start codon in both tribes, followed by ATA, which was examined in all species; ATT and ATC were rarely used (Fig 5A). For stop codons, TAA was the most frequent stop codon, and incomplete stop codons (TA-/T--) were more common than the complete codons TAG, AGG, and AGA (Fig 5B).

**Fig 6:**
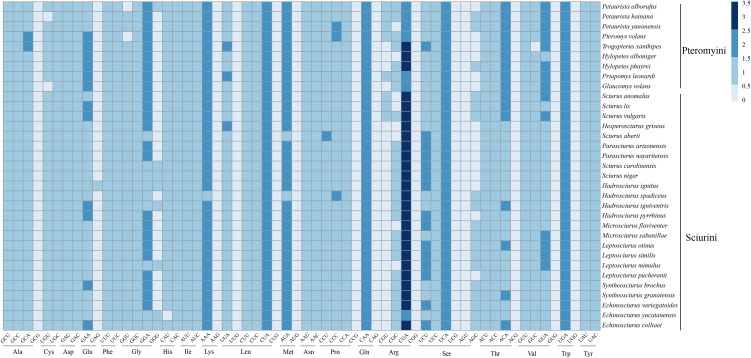
Relative synonymous codon usage (RSCU) in Pteromyini and Sciurini.

**Fig 7:**
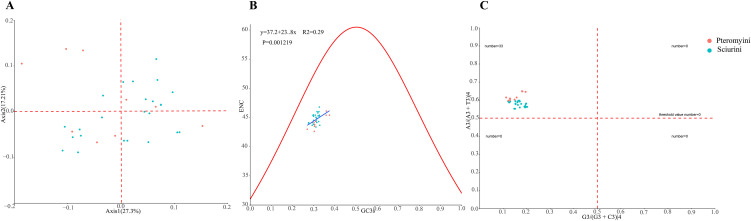
Analysis of codon usage bias in Pteromyini and Sciurini. (A) COA, (B) ENC-Plot, (C) PR2-Plot.

The relative synonymous codon usage (RSCU) analysis showed that each species of Pteromyini had 30 highly expressed codons (RSCU > 1), while each species of Sciurini had 31 highly expressed codons. Both tribes exhibited high usage frequency for UGA, UCA, CGA, CAA, AUA, CUA, AAA, and GGA. Additionally, Pteromyini showed higher usage frequencies for codons ACA, GUA, and GAA, whereas Sciurini had higher usage for UCU and showed a marked preference for CGA (Fig 6). Correspondence analysis (COA) revealed distinct codon usage bias between the two tribes. Species within the Pteromyini and Sciurini tribes exhibit intratribal variation, distributed widely along two axes. However, the Pteromyini tribe has multiple outliers, whereas the Sciurini tribe has very few outliers, indicating a more conservative codon usage bias within the tribe (Fig 7A). The ENC-plot further characterized that bias (Fig 7B). Both tribes exhibited high ENC values (Pteromyini: 42.63 to 46.11; Sciurini: 43.37 to 46.80) and corresponding with GC3s contents (Pteromyini: 0.361 to 0.417; Sciurini: 0.364 to 0.400), both tribes exhibited high ENC values and a weak codon usage bias. The points for species of both tribes were located below the standard curve, indicating that natural selection was an influencing factor for codon usage bias (Fig 7B). In the neutrality plot (with four quadrants centered at 0.5), all species were located in the first quadrant (A3/(A3 + T3)4 > 0.50 and G3/(G3 + C3)4 < 0.50), revealing that the third codon position followed the pattern of A > T and C > G, exhibiting an AC bias. No species was located at the center point (0.5, 0.5), which further indicated that codon usage bias in both tribes was mainly influenced by natural selection (Fig 7C).

### 3.5. Phylogenetic analysis

**Fig 8:**
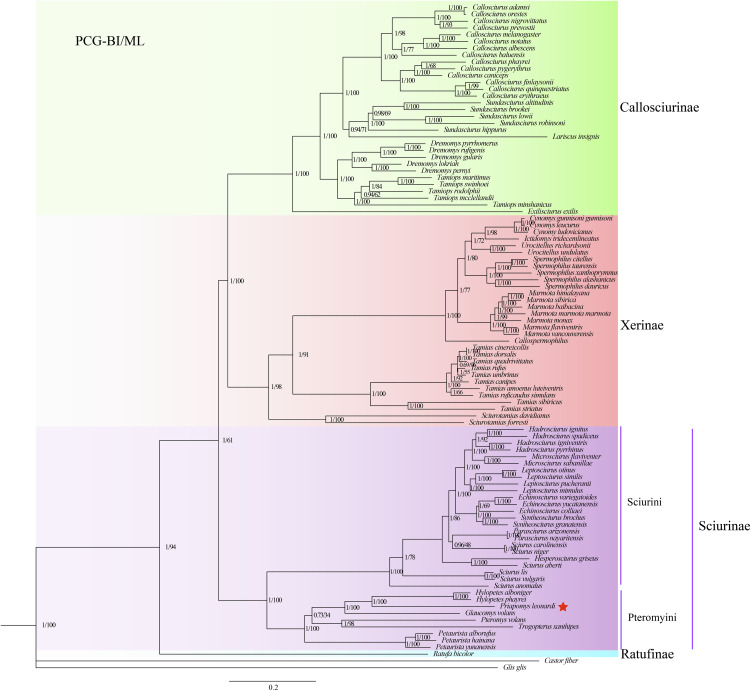
Phylogenetic relationships of the Sciuridae inferred from Bayesian inference (BI) and maximum likelihood (ML) analysis of 13 protein-coding genes (PCG). Node labels show the posterior probability (PP)/bootstrap support (BS) (%) values. *Priapomys leonardi* is marked by a red pentagram.

We reconstructed the phylogeny using two concatenated datasets (PCG and PCGRNA) and two tree-building methods (ML and BI). The phylogenetic trees obtained from different methods and datasets shared the same topology (Fig 8, S1 Fig). Phylogenetic relationship among the subfamilies of Sciuridae was ((Callosciurinae + Xerinae) + Sciurinae) + Ratufinae. Within Callosciurinae, phylogenetic relationship was *Exilisciurus* + ((*Tamiops* + *Dremomys*) + ((*Lariscus* + *Sundasciurus*) + *Callosciurus*)). Within Xerinae, phylogenetic relationship was *Sciurotamias* + (*Tamias* + (*Callospermophilus* + (*Marmota* + (*Spermophilus* + (*Urocitellus* + (*Ictidomys* + *Cynomys*)))))). Within Sciurinae, phylogenetic relationship was ((*Glaucomys* + (*Hylopetes* + *Priapomys*)) + ((*Trogopterus* + *Pteromys*) + *Petaurista*) + *Sciurus*). Phylogenetic trees supported the sister relationship between Pteromyini and Sciurini, which together formed the subfamily Sciurinae, with high nodal support (PP = 1/BS = 100). Specifically, *Priapomys leonardi* was identified as a distinct lineage, forming a sister clade with *Hylopetes*, indicating its closest relationship to *Hylopetes*. Finally, the clade comprising *Priapomys leonardi* and *Hylopetes* was found to be sister to *Glaucomys* of the only North American clade.

## 4. Discussion

As two tribes within the subfamily Sciurinae, Pteromyini and Sciurini share high similarities in certain physiological traits (e.g., dentition) and taxonomic classification. However, adaptation to different ecological niches has driven divergence in their locomotor modes, circadian rhythms, and body morphology. The presence or absence of a patagium (gliding membrane) in Pteromyini fundamentally dictates their locomotor modes and lifestyle, leading to pronounced morphological disparity. Pteromyini are nocturnal gliders and exhibit a broad spectrum of body and tail lengths——ranging from the smallest species, Glaucomys volans (Linnaeus 1758) (body length 117 mm, tail length 80 mm), to the largest species, Petaurista yunanensis (Anderon,1875) (body length 610 mm, tail length 605 mm). This extensive morphological variation indicates high disparity, broad habitat adaptability, and occupancy of a wider ecological niche [32,33]. In contrast, Sciurini exhibit a relatively narrow and uniform range of body tail dimensions (Table 1).

The mitogenomes of both Pteromyini and Sciurini were approximately 16 kb in size (S1 Table), and shared identical gene arrangement and composition (Fig 1). This mitogenomic organization was highly conserved and conformed to the typical pattern of both Sciuridae [34] and Rodentia [35]. Notably, these mitogenomes showed no evidence of gene rearrangement or fragmentation events of the type observed in some arthropods [36].

A comparative analysis of mitogenomes of Pteromyini and Sciurini shared highly conserved feature, including AT content, base composition at codon site, and usage of start and stop codons. Specifically, AT content was slightly higher than GC content (Fig 2), and was the highest at third codon position (Fig 3); ATG and TAA were the most predominant start and stop codons, respectively (Fig 5). These findings indicated that divergent locomotor modes have had minimal impact on these fundamental mitogenomic features. In mammals, AT-skew is generally positive and GC-skew is negative [37]. While all Pteromyini species conformed to this pattern, two Sciurini species (*Parasciurus arizonensis* and *Hadrosciurus ignitus*) exhibited a slightly negative AT-skew (−0.003). For all mitochondrial protein-coding genes in both tribes, Ka, Ks, and Ka/Ks values were less than 1 (S2 Table), indicating that purifying selection plays a central role in evolution of mitochondrial DNA, preserving its critical function in energy metabolism [38]. Among these genes, *ATP8* showed the highest Ka/Ks ratio, whereas *COX1* had the lowest, indicating the fastest and the slowest evolutionary rates, respectively——a pattern also reported in other rodents [39] that may be related to the shorter sequence length and lower functional constraints of *ATP8*. This result is also consistent with the condlusion of Tu et al., who investigated evolutionary rates of mitochondrial protein-coding genes in the flying squirrel and squirrel families using different methods [40]. Notably, the average Ka/Ks ratios for *ATP8*, *COX1*, *CYTB*, and *ND5* were higher in Pteromyini than in Sciurini (Fig 4), which was primarily driven by their higher non-synonymous mutation rates (Ka) (S2 Table). The relatively higher (though still <1) Ka/Ks ratios in Pteromyini as evidence of relaxed purifying selection or reduced evolutionary constraint compared to Sciurini. This interpretation is consistent with the observation that all genes are under purifying selection (Ka/Ks < 1), while still allowing for lineage-specific differences in the stringency of selection. A correlation between mitogenomic evolution and locomotor modes has been documented in birds and bats, where positive selection was often reported [41,42]. In contrast, studies in rodents (Sciuridae and Muridae) consistently highlight the dominance of purifying selection [39,43]. This discrepancy suggested that different taxonomic groups may experience distinct selective pressures during evolution.

Relative Synonymous Codon Usage (RSCU) measures the relative probability of specific codons being used among its synonymous alternatives for a given amino acids [44]. RSCU analysis indicated that both Pteromyini and Sciurini exhibited high usage frequencies for eight codons (UGA, UCA, CGA, CAA, AUA, CUA, AAA, GGA). Notably, however, the gliding Pteromyini showed a high frequencies for three additional codons (ACA, GUA, GAA) compared to Sciurini, whereas the scansorial Sciurini displayed a particular preference for CGA (Fig 6). To further investigate codon usage patterns, we performed correspondence analysis (COA), a multivariate method that projects RSCU of each species into a multidimensional space (with 60 dimensions corresponding to [45,46]. COA revealed variations in codon usage within the gliding Pteromyini, whereas the scansorial Sciurini exhibited a more conserved codon usage. This indicates there are differences in codon usage between clades with different locomotor modes (Fig 7A). In addition, the Effective Number of Codons (ENC) ranges from 20 (extreme bias) to 61 (no bias), with lower values indicating codon usage bias (significant codon bias is typically considered when ENC ≤ 35) [47]. Parity Rule 2 (PR2) analysis was used to evaluate the influence of selection and mutation by examining AT and GC biases at the third position [48]. Both ENC-plot analysis and PR2 analyses suggested that natural selection has shaped codon usage in both tribes (Fig 7B, C). This implies that clades with different locomotor modes may nonetheless experience similar selective pressures during evolution.

We reconstructed the phylogeny of the family Sciuridae using two phylogenetic methods (ML and BI) and two concatenated datasets (PCG and PCGRNA). The four phylogenetic trees were fully congruent in their topologies (Fig 8, S1 Fig). Our analysis robustly revealed four monophyletic subfamilies: Ratufinae, Sciurinae, Xerinae, and Callosciurinae. The phylogenetic relationship among these subfamilies was ((Callosciurinae + Xerinae) + Sciurinae) + Ratufinae, with high nodal support. These results were mostly consistent with previous studies [49], in confirming the monophyly of each subfamily. However, unlike the findings of Casanovas-Vilar et al. [50], our analysis supports a sister-group relationship between Callosciurinae and Xerinae. Meanwhile, our phylogeny contradicts the result of Mercer and Roth (2003) [7] who treated Xerinae and Sciurinae as sister groups, and is consistent with Steppan et al. (2004) [8].

## 5. Conclusion

This study presented a comparative mitogenomic analysis between the gliding tribe Pteromyini and the scansorial tribe Sciurini, incorporating newly sequenced data from *Priapomys leonardi* with publicly available data. The mitogenomes of both tribes were highly conserved, approximately 16 kb in size with identical gene order conforming to the typical rodent pattern. For all protein-coding genes in both tribes, Ka/Ks values were much less than 1 (Fig 4, S2 Table), providing support evidence for purifying selection. However, the average Ka/Ks ratios of *ATP8*, *COX1*, *CYTB*, and *ND5* genes were higher in the gliding Pteromyini were higher than in the scansorial Sciurini. Codon usage bias analysis revealed greater variation within Pteromyini, whereas Sciurini exhibited higher conservation. Both tribes were affected by natural selection during their evolutionary processes. Phylogenetic analysis robustly revealed four monophyletic subfamilies: Ratufinae, Sciurinae, Xerinae, and Callosciurinae. The phylogenetic relationship among these subfamilies was ((Callosciurinae + Xerinae) + Sciurinae) + Ratufinae, with high nodal support. Phylogenetic reconstruction placed *Priapomys leonardi* within Sciuridae, and supported a sister relationship between Pteromyini (flying squirrels) and Sciurini (tree squirrels). Our analysis supporting a sister-group relationship between Callosciurinae and Xerinae. Within Sciurinae, Pteromyini and Sciurini were recovered as sister tribes and supported that the gliding Pteromyini evolved from the scansorial Sciurini ancestors. This study provides insights into locomotor modes and evolutionary relationships of the family Sciuridae.

## Supporting information

S1 Table Species information. (DOCX)

S2 Table The Ka, Ks, Ka/Ks values of protein-coding genes in Pteromyini and Sciurini. (DOCX)

S1 Fig Phylogenetic relationships of the Sciuridae inferred from Bayesian inference (BI) and maximum likelihood (ML) analysis of 13 protein-coding genes and rRNAs (PCGRNA). Node labels show the posterior probability (PP)/bootstrap support (BS) (%) values. Priapomys leonardi is marked by a red pentagram.
(TIF)
