# Supplementary material for: Insights into adaptive evolution in different locomotor modes and phylogeny of Sciuridae from mitogenomes of flying and tree squirrels
Source: PLoS One. 2026 Aug 14;21(8):e0355043. doi: 10.1371/journal.pone.0355043 (PMC13475863; doi:10.1371/journal.pone.0355043)
Supplement: Supplementary file 1 [file pone.0355043.s001.docx]

| **Table S1. Species information.** | | | | | | | |
| --- | --- | --- | --- | --- | --- | --- | --- |
|  | Family | Subfamily | Tribe | Genus | Species | GenBank  number | Size (bp) |
| Outgroup | Gliridae | Glirinae |  | *Glis* | *Glis glis* | NC001892 | 16,602 |
|  | Castoridae |  |  | *Castor* | *Castor fiber* | NC028625 | 16,722 |
| Ingroup | Sciuridae | Ratufinae |  | *Ratufa* | *Ratufa bicolor* | NC023780 | 16,563 |
|  |  | Sciurinae | Pteromyini | *Glaucomys* | *Glaucomys volans* | NC050026 | 16,535 |
|  |  |  |  | *Hylopetes* | *Hylopetes alboniger* | NC031847 | 16,584 |
|  |  |  |  |  | *Hylopetes phayrei* | NC026443 | 16,530 |
|  |  |  |  | *Petaurista* | *Petaurista alborufus* | MN011572 | 16,511 |
|  |  |  |  |  | *Petaurista hainana* | NC023089 | 16,502 |
|  |  |  |  |  | *Petaurista yunanensis* | NC033902 | 16,620 |
|  |  |  |  | *Pteromys* | *Pteromys volans* | NC019612 | 16,513 |
|  |  |  |  | *Trogoptreus* | *Trogopterus xanthipes* | PQ280108 | 16,529 |
|  |  |  | Sciurini | *Sciurus* | *Sciurus aberti* | MT211954 | 16,504 |
|  |  |  |  |  | *Sciurus anomalus* | ON620284 | 16,510 |
|  |  |  |  |  | *Sciurus carolinensis* | NC050012 | 16,516 |
|  |  |  |  |  | *Sciurus lis* | MT134013 | 16,512 |
|  |  |  |  |  | *Sciurus niger* | KY411003 | 16,164 |
|  |  |  |  |  | *Sciurus vulgaris* | NC002369 | 16,507 |
|  |  |  |  |  | *Syntheosciurus brochus* | MT240890 | 16,523 |
|  |  |  |  |  | *Syntheosciurus granatensis* | NC050018 | 16,515 |
|  |  |  |  |  | *Echinosciurus colliaei* | NC050013 | 16,519 |
|  |  |  |  |  | *Echinosciurus variegatoides* | NC050015 | 16,521 |
|  |  |  |  |  | *Echinosciurus yucatanensis* | NC050016 | 16,519 |
|  |  |  |  |  | *Hadrosciurus igniventris* | NC050027 | 16,518 |
|  |  |  |  |  | *Hadrosciurus pyrrhinus* | NC050028 | 16,512 |
|  |  |  |  |  | *Hadrosciurus spadiceus* | NC050029 | 16,515 |
|  |  |  |  |  | *Hadrosciurus ignitus* | NC050032 | 16,519 |
|  |  |  |  |  | *Hesperosciurus griseus* | NC050034 | 16,520 |
|  |  |  |  |  | *Leptosciurus pucheranii* | NC050017 | 16,504 |
|  |  |  |  |  | *Leptosciurus mimulus* | NC050020 | 16,504 |
|  |  |  |  |  | *Leptosciurus otinus* | NC050021 | 16,516 |
|  |  |  |  |  | *Leptosciurus similis* | NC050022 | 16,520 |
|  |  |  |  |  | *Microsciurus flaviventer* | NC050030 | 16,521 |
|  |  |  |  |  | *Microsciurus sabanillae* | NC050031 | 16,524 |
|  |  |  |  |  | *Parasciurus arizonensis* | NC050011 | 16,519 |
|  |  |  |  |  | *Parasciurus nayaritensis* | NC050014 | 16,521 |
|  |  | Callosciurinae |  | *Callosciurus* | *Callosciurus erythraeus* | KP708709 | 16,550 |
|  |  |  |  |  | *Callosciurus finlaysonii* | NC035817 | 16,490 |
|  |  |  |  |  | *Callosciurus prevostii* | NC035816 | 16,674 |
|  |  |  |  |  | *Callosciurus pygerythrus* | KY410913 | 16,175 |
|  |  |  |  |  | *Callosciurus albescens* | KY410906 | 16,222 |
|  |  |  |  |  | *Callosciurus melanogaster* | KY410908 | 16,254 |
|  |  |  |  |  | *Callosciurus nigrovittatus* | KY410866 | 16,181 |
|  |  |  |  |  | *Callosciurus baluensis* | KY410847 | 16,182 |
|  |  |  |  |  | *Callosciurus orestes* | KY410869 | 16,179 |
|  |  |  |  |  | *Callosciurus adamsi* | NC030071 | 15,613 |
|  |  |  |  |  | *Callosciurus notatus* | MT231329 | 16,582 |
|  |  |  |  |  | *Callosciurus quinquestriatus* | KY410891 | 16,178 |
|  |  |  |  |  | *Callosciurus phayrei* | KY410851 | 16,166 |
|  |  |  |  |  | *Callosciurus caniceps* | KY410858 | 16,174 |
|  |  |  |  | *Dremomys* | *Dremomys rufigenis* | NC026442 | 16,545 |
|  |  |  |  |  | *Dremomys pernyi* | NC035577 | 16,573 |
|  |  |  |  |  | *Dremomys pyrrhomerus* | KP708705 | 16,549 |
|  |  |  |  |  | *Dremomys lokriah* | KY410879 | 16,166 |
|  |  |  |  |  | *Dremomys gularis* | OQ160785 | 16,629 |
|  |  |  |  | *Tamiops* | *Tamiops swinhoei* | NC026875 | 16,513 |
|  |  |  |  |  | *Tamiops maritimus* | NC029325 | 16,523 |
|  |  |  |  |  | *Tamiops mcclellandii* | NC080357 | 16,629 |
|  |  |  |  |  | *Tamiops rodolphii* | KY410983 | 16,137 |
|  |  |  |  |  | *Tamiops minshanicus* | PQ591919 | 16,559 |
|  |  |  |  | *Exilisciurus* | *Exilisciurus exilis* | NC030072 | 16,337 |
|  |  |  |  | *Lariscus* | *Lariscus insignis* | NC030070 | 16,355 |
|  |  |  |  | *Sundasciurus* | *Sundasciurus brookei* | NC035812 | 16,418 |
|  |  |  |  |  | *Sundasciurus altitudinis* | MT157213 | 16,308 |
|  |  |  |  |  | *Sundasciurus robinsoni* | MT157214 | 16,324 |
|  |  |  |  |  | *Sundasciurus lowii* | MT157215 | 16,309 |
|  |  |  |  |  | *Sundasciurus hippurus* | MT157216 | 16,229 |
|  |  | Xerinae | Marmotini | *Marmota* | *Marmota marmota marmota* | MN935776 | 16,434 |
|  |  |  |  |  | *Marmota monax* | JAMOFY010003380 | 16,439 |
|  |  |  |  |  | *Marmota flaviventris* | NC042243 | 16,438 |
|  |  |  |  |  | *Marmota himalayana* | NC018367 | 16,443 |
|  |  |  |  |  | *Marmota vancouverensis* | NC048490 | 16,435 |
|  |  |  |  |  | *Marmota baibacina* | NC086592 | 16,444 |
|  |  |  |  |  | *Marmota sibirica* | NC086593 | 16,437 |
|  |  |  |  | *Spermophilus* | *Spermophilus alashanicus* | NC071768 | 16,464 |
|  |  |  |  |  | *Spermophilus citellus* | PQ533849 | 16,449 |
|  |  |  |  |  | *Spermophilus dauricus* | KP708706 | 16,487 |
|  |  |  |  |  | *Spermophilus taurensis* | OQ675160 | 16,447 |
|  |  |  |  |  | *Spermophilus xanthoprymnus* | PQ533851 | 16,469 |
|  |  |  |  | *Sciurotamias* | *Sciurotamias forresti* | PP175169 | 16,534 |
|  |  |  |  |  | *Sciurotamias davidianus* | KP708708 | 16,570 |
|  |  |  |  | *Tamias* | *Tamias sibiricus* | NC025277 | 16,558 |
|  |  |  |  |  | *Tamias quadrivittatus* | NC032370 | 16,549 |
|  |  |  |  |  | *Tamias rufus* | NC032371 | 16,543 |
|  |  |  |  |  | *Tamias canipes* | NC032372 | 16,552 |
|  |  |  |  |  | *Tamias dorsalis* | NC032373 | 16,549 |
|  |  |  |  |  | *Tamias cinereicollis* | NC032374 | 16,549 |
|  |  |  |  |  | *Tamias striatus* | NC032375 | 16,553 |
|  |  |  |  |  | *Tamias umbrinus* | NC032376 | 16,552 |
|  |  |  |  |  | *Tamias ruficaudus simulans* | KY070177 | 16,553 |
|  |  |  |  |  | *Tamias amoenus luteiventris* | KY070157 | 16,545 |
|  |  |  |  | *Callospermophilus* | *Callospermophilus lateralis* | NC031210 | 16,457 |
|  |  |  |  | *Cynomys* | *Cynomys ludovicianus* | KP326310 | 16,466 |
|  |  |  |  |  | *Cynomys leucurus* | KP326309 | 16,454 |
|  |  |  |  |  | *Cynomys gunnisoni gunnisoni* | MG450794 | 16,461 |
|  |  |  |  | *Ictidomys* | *Ictidomys tridecemlineatus* | KP698974 | 16,458 |
|  |  |  |  | *Urocitellus* | *Urocitellus richardsonii* | NC031209 | 16,460 |
|  |  |  |  |  | *Urocitellus undulatus* | PQ720778 | 16,456 |
