# Supplementary material for: Insights into adaptive evolution in different locomotor modes and phylogeny of Sciuridae from mitogenomes of flying and tree squirrels
Source: PLoS One. 2026 Aug 14;21(8):e0355043. doi: 10.1371/journal.pone.0355043 (PMC13475863; doi:10.1371/journal.pone.0355043)
Supplement: Supplementary file 2 [file pone.0355043.s002.docx]

| **Table S2. The Ka, Ks, Ka/Ks values of protein-coding genes in Pteromyini and Sciurini.** | | | | | | | | | | | | | | |
| --- | --- | --- | --- | --- | --- | --- | --- | --- | --- | --- | --- | --- | --- | --- |
| Tribe | Species | Ka | | | | | | | | | | | | |
|  |  | *ND1* | *ND2* | *COX1* | *COX2* | *ATP8* | *ATP6* | *COX3* | *ND3* | *ND4L* | *ND4* | *ND5* | *ND6* | *CYTB* |
| Pteromyini | Petaurista alborufus | 0.137 | 0.227 | 0.052 | 0.055 | 0.267 | 0.095 | 0.075 | 0.160 | 0.136 | 0.159 | 0.157 | 0.156 | 0.079 |
|  | Petaurista hainana | 0.139 | 0.229 | 0.053 | 0.051 | 0.263 | 0.097 | 0.072 | 0.161 | 0.132 | 0.158 | 0.158 | 0.163 | 0.076 |
|  | Petaurista yunanensis | 0.137 | 0.225 | 0.053 | 0.055 | 0.312 | 0.091 | 0.074 | 0.149 | 0.148 | 0.163 | 0.157 | 0.161 | 0.074 |
|  | Pteromys volans | 0.120 | 0.227 | 0.053 | 0.057 | 0.289 | 0.101 | 0.074 | 0.142 | 0.142 | 0.155 | 0.164 | 0.153 | 0.070 |
|  | Trogopterus xanthipes | 0.138 | 0.246 | 0.056 | 0.064 | 0.241 | 0.096 | 0.077 | 0.186 | 0.174 | 0.155 | 0.158 | 0.210 | 0.081 |
|  | Hylopetes alboniger | 0.125 | 0.215 | 0.052 | 0.060 | 0.264 | 0.100 | 0.078 | 0.158 | 0.154 | 0.155 | 0.168 | 0.166 | 0.080 |
|  | Hylopetes phayrei | 0.125 | 0.236 | 0.057 | 0.074 | 0.261 | 0.100 | 0.078 | 0.162 | 0.155 | 0.158 | 0.165 | 0.167 | 0.082 |
|  | Priapomys leonardi | 0.125 | 0.236 | 0.054 | 0.061 | 0.248 | 0.106 | 0.083 | 0.149 | 0.153 | 0.162 | 0.175 | 0.159 | 0.084 |
|  | Glaucomys volans | 0.132 | 0.238 | 0.053 | 0.059 | 0.256 | 0.091 | 0.082 | 0.174 | 0.131 | 0.156 | 0.153 | 0.161 | 0.074 |
| Sciurini | Sciurus anomalus | 0.125 | 0.244 | 0.050 | 0.059 | 0.263 | 0.119 | 0.079 | 0.177 | 0.182 | 0.157 | 0.163 | 0.134 | 0.064 |
|  | Sciurus lis | 0.133 | 0.246 | 0.046 | 0.060 | 0.261 | 0.087 | 0.077 | 0.159 | 0.181 | 0.173 | 0.158 | 0.142 | 0.063 |
|  | Sciurus vulgaris | 0.129 | 0.241 | 0.048 | 0.062 | 0.264 | 0.094 | 0.076 | 0.155 | 0.181 | 0.172 | 0.158 | 0.147 | 0.063 |
|  | Hesperosciurus griseus | 0.132 | 0.247 | 0.049 | 0.059 | 0.248 | 0.105 | 0.079 | 0.159 | 0.195 | 0.158 | 0.166 | 0.150 | 0.074 |
|  | Sciurus aberti | 0.133 | 0.246 | 0.050 | 0.061 | 0.246 | 0.096 | 0.075 | 0.153 | 0.194 | 0.164 | 0.162 | 0.149 | 0.078 |
|  | Parasciurus arizonensis | 0.134 | 0.299 | 0.055 | 0.062 | 0.262 | 0.104 | 0.080 | 0.169 | 0.178 | 0.161 | 0.155 | 0.138 | 0.071 |
|  | Parasciurus nayaritensis | 0.134 | 0.247 | 0.055 | 0.062 | 0.264 | 0.104 | 0.081 | 0.169 | 0.183 | 0.163 | 0.153 | 0.138 | 0.073 |
|  | Sciurus carolinensis | 0.134 | 0.247 | 0.050 | 0.063 | 0.237 | 0.096 | 0.082 | 0.168 | 0.196 | 0.158 | 0.167 | 0.143 | 0.075 |
|  | Sciurus niger | 0.133 | 0.245 | 0.050 | 0.061 | 0.237 | 0.096 | 0.080 | 0.168 | 0.196 | 0.158 | 0.169 | 0.143 | 0.075 |
|  | Hadrosciurus ignitus | 0.130 | 0.238 | 0.050 | 0.053 | 0.239 | 0.103 | 0.079 | 0.152 | 0.204 | 0.157 | 0.168 | 0.133 | 0.074 |
|  | Hadrosciurus spadiceus | 0.133 | 0.239 | 0.052 | 0.054 | 0.257 | 0.097 | 0.081 | 0.160 | 0.187 | 0.154 | 0.163 | 0.136 | 0.076 |
|  | Hadrosciurus igniventris | 0.127 | 0.236 | 0.050 | 0.053 | 0.224 | 0.090 | 0.081 | 0.159 | 0.187 | 0.158 | 0.158 | 0.128 | 0.080 |
|  | Hadrosciurus pyrrhinus | 0.131 | 0.239 | 0.053 | 0.052 | 0.215 | 0.090 | 0.075 | 0.164 | 0.190 | 0.155 | 0.156 | 0.127 | 0.074 |
|  | Microsciurus flaviventer | 0.131 | 0.246 | 0.051 | 0.052 | 0.212 | 0.099 | 0.079 | 0.165 | 0.187 | 0.159 | 0.161 | 0.137 | 0.083 |
|  | Microsciurus sabanillae | 0.126 | 0.252 | 0.052 | 0.064 | 0.246 | 0.096 | 0.083 | 0.161 | 0.186 | 0.161 | 0.164 | 0.136 | 0.083 |
|  | Leptosciurus otinus | 0.130 | 0.246 | 0.053 | 0.064 | 0.242 | 0.101 | 0.081 | 0.159 | 0.171 | 0.167 | 0.151 | 0.139 | 0.076 |
|  | Leptosciurus similis | 0.131 | 0.246 | 0.056 | 0.062 | 0.239 | 0.101 | 0.070 | 0.158 | 0.178 | 0.160 | 0.152 | 0.141 | 0.073 |
|  | Leptosciurus mimulus | 0.125 | 0.247 | 0.052 | 0.057 | 0.259 | 0.099 | 0.089 | 0.161 | 0.194 | 0.159 | 0.159 | 0.143 | 0.078 |
|  | Leptosciurus pucheranii | 0.131 | 0.240 | 0.053 | 0.067 | 0.231 | 0.096 | 0.075 | 0.155 | 0.199 | 0.159 | 0.159 | 0.142 | 0.079 |
|  | Syntheosciurus brochus | 0.125 | 0.250 | 0.050 | 0.060 | 0.239 | 0.101 | 0.075 | 0.164 | 0.187 | 0.168 | 0.159 | 0.155 | 0.092 |
|  | Syntheosciurus granatensis | 0.126 | 0.240 | 0.049 | 0.057 | 0.239 | 0.099 | 0.077 | 0.175 | 0.191 | 0.163 | 0.160 | 0.143 | 0.073 |
|  | Echinosciurus variegatoides | 0.129 | 0.236 | 0.048 | 0.062 | 0.235 | 0.094 | 0.076 | 0.163 | 0.184 | 0.163 | 0.158 | 0.147 | 0.069 |
|  | Echinosciurus yucatanensis | 0.125 | 0.240 | 0.050 | 0.066 | 0.233 | 0.101 | 0.078 | 0.162 | 0.178 | 0.163 | 0.160 | 0.149 | 0.071 |
|  | Echinosciurus colliaei | 0.129 | 0.232 | 0.051 | 0.060 | 0.243 | 0.095 | 0.081 | 0.163 | 0.174 | 0.163 | 0.161 | 0.144 | 0.074 |
| Tribe | Species | Ks | | | | | | | | | | | | |
|  |  | *ND1* | *ND2* | *COX1* | *COX2* | *ATP8* | *ATP6* | *COX3* | *ND3* | *ND4L* | *ND4* | *ND5* | *ND6* | *CYTB* |
| Pteromyini | Petaurista alborufus | 0.691 | 0.614 | 0.687 | 0.746 | 0.626 | 0.702 | 0.621 | 0.746 | 0.674 | 0.691 | 0.631 | 0.646 | 0.672 |
|  | Petaurista hainana | 0.656 | 0.624 | 0.696 | 0.772 | 0.598 | 0.693 | 0.629 | 0.653 | 0.724 | 0.713 | 0.659 | 0.679 | 0.714 |
|  | Petaurista yunanensis | 0.709 | 0.663 | 0.716 | 0.699 | 0.558 | 0.711 | 0.645 | 0.684 | 0.764 | 0.719 | 0.663 | 0.686 | 0.692 |
|  | Pteromys volans | 0.683 | 0.670 | 0.655 | 0.681 | 0.642 | 0.735 | 0.622 | 0.705 | 0.707 | 0.673 | 0.667 | 0.634 | 0.731 |
|  | Trogopterus xanthipes | 0.665 | 0.665 | 0.628 | 0.671 | 0.524 | 0.655 | 0.595 | 0.664 | 0.681 | 0.673 | 0.620 | 0.529 | 0.717 |
|  | Hylopetes alboniger | 0.669 | 0.644 | 0.645 | 0.709 | 0.489 | 0.704 | 0.581 | 0.701 | 0.682 | 0.676 | 0.649 | 0.588 | 0.680 |
|  | Hylopetes phayrei | 0.669 | 0.644 | 0.637 | 0.747 | 0.610 | 0.684 | 0.574 | 0.683 | 0.634 | 0.685 | 0.657 | 0.579 | 0.696 |
|  | Priapomys leonardi | 0.648 | 0.644 | 0.615 | 0.715 | 0.563 | 0.688 | 0.652 | 0.706 | 0.667 | 0.703 | 0.685 | 0.648 | 0.691 |
|  | Glaucomys volans | 0.650 | 0.601 | 0.698 | 0.670 | 0.424 | 0.648 | 0.617 | 0.599 | 0.630 | 0.714 | 0.678 | 0.595 | 0.668 |
| Sciurini | Sciurus anomalus | 0.672 | 0.668 | 0.726 | 0.643 | 0.685 | 0.716 | 0.694 | 0.687 | 0.733 | 0.704 | 0.657 | 0.642 | 0.743 |
|  | Sciurus lis | 0.653 | 0.685 | 0.729 | 0.745 | 0.566 | 0.697 | 0.605 | 0.771 | 0.711 | 0.677 | 0.642 | 0.578 | 0.765 |
|  | Sciurus vulgaris | 0.663 | 0.693 | 0.709 | 0.748 | 0.599 | 0.717 | 0.613 | 0.753 | 0.724 | 0.656 | 0.659 | 0.573 | 0.765 |
|  | Hesperosciurus griseus | 0.666 | 0.625 | 0.717 | 0.686 | 0.601 | 0.672 | 0.657 | 0.639 | 0.691 | 0.737 | 0.683 | 0.568 | 0.723 |
|  | Sciurus aberti | 0.679 | 0.698 | 0.718 | 0.679 | 0.535 | 0.649 | 0.647 | 0.721 | 0.698 | 0.701 | 0.767 | 0.652 | 0.746 |
|  | Parasciurus arizonensis | 0.633 | 0.659 | 0.704 | 0.648 | 0.607 | 0.667 | 0.645 | 0.652 | 0.628 | 0.732 | 0.677 | 0.589 | 0.748 |
|  | Parasciurus nayaritensis | 0.629 | 0.694 | 0.699 | 0.648 | 0.605 | 0.673 | 0.644 | 0.664 | 0.625 | 0.725 | 0.686 | 0.595 | 0.761 |
|  | Sciurus carolinensis | 0.686 | 0.726 | 0.699 | 0.694 | 0.580 | 0.720 | 0.669 | 0.721 | 0.769 | 0.676 | 0.668 | 0.560 | 0.694 |
|  | Sciurus niger | 0.682 | 0.717 | 0.705 | 0.699 | 0.602 | 0.714 | 0.668 | 0.697 | 0.783 | 0.688 | 0.670 | 0.574 | 0.698 |
|  | Hadrosciurus ignitus | 0.715 | 0.702 | 0.738 | 0.726 | 0.600 | 0.684 | 0.721 | 0.734 | 0.688 | 0.749 | 0.695 | 0.602 | 0.725 |
|  | Hadrosciurus spadiceus | 0.697 | 0.685 | 0.721 | 0.652 | 0.609 | 0.678 | 0.637 | 0.707 | 0.641 | 0.729 | 0.710 | 0.620 | 0.726 |
|  | Hadrosciurus igniventris | 0.671 | 0.663 | 0.698 | 0.726 | 0.562 | 0.660 | 0.669 | 0.731 | 0.711 | 0.680 | 0.689 | 0.609 | 0.739 |
|  | Hadrosciurus pyrrhinus | 0.642 | 0.645 | 0.714 | 0.677 | 0.589 | 0.660 | 0.619 | 0.734 | 0.738 | 0.673 | 0.691 | 0.565 | 0.733 |
|  | Microsciurus flaviventer | 0.666 | 0.671 | 0.705 | 0.729 | 0.706 | 0.625 | 0.657 | 0.726 | 0.678 | 0.731 | 0.740 | 0.590 | 0.718 |
|  | Microsciurus sabanillae | 0.673 | 0.698 | 0.728 | 0.713 | 0.528 | 0.689 | 0.610 | 0.702 | 0.687 | 0.704 | 0.651 | 0.605 | 0.719 |
|  | Leptosciurus otinus | 0.685 | 0.650 | 0.728 | 0.710 | 0.566 | 0.711 | 0.661 | 0.692 | 0.624 | 0.685 | 0.644 | 0.513 | 0.754 |
|  | Leptosciurus similis | 0.646 | 0.660 | 0.717 | 0.677 | 0.627 | 0.713 | 0.650 | 0.658 | 0.650 | 0.710 | 0.670 | 0.557 | 0.729 |
|  | Leptosciurus mimulus | 0.705 | 0.690 | 0.679 | 0.683 | 0.620 | 0.706 | 0.608 | 0.708 | 0.731 | 0.744 | 0.644 | 0.569 | 0.748 |
|  | Leptosciurus pucheranii | 0.631 | 0.613 | 0.719 | 0.637 | 0.602 | 0.721 | 0.690 | 0.715 | 0.717 | 0.734 | 0.659 | 0.584 | 0.744 |
|  | Syntheosciurus brochus | 0.698 | 0.628 | 0.718 | 0.716 | 0.670 | 0.642 | 0.691 | 0.731 | 0.761 | 0.684 | 0.672 | 0.584 | 0.735 |
|  | Syntheosciurus granatensis | 0.696 | 0.657 | 0.716 | 0.700 | 0.668 | 0.641 | 0.659 | 0.683 | 0.785 | 0.707 | 0.664 | 0.550 | 0.702 |
|  | Echinosciurus variegatoides | 0.685 | 0.675 | 0.703 | 0.693 | 0.644 | 0.683 | 0.680 | 0.790 | 0.773 | 0.706 | 0.682 | 0.543 | 0.790 |
|  | Echinosciurus yucatanensis | 0.651 | 0.688 | 0.736 | 0.641 | 0.593 | 0.689 | 0.716 | 0.783 | 0.720 | 0.727 | 0.672 | 0.585 | 0.715 |
|  | Echinosciurus colliaei | 0.675 | 0.676 | 0.677 | 0.678 | 0.697 | 0.679 | 0.671 | 0.797 | 0.771 | 0.756 | 0.709 | 0.565 | 0.687 |
| Tribe | Species | Ka/Ks | | | | | | | | | | | | |
|  |  | *ND1* | *ND2* | *COX1* | *COX2* | *ATP8* | *ATP6* | *COX3* | *ND3* | *ND4L* | *ND4* | *ND5* | *ND6* | *CYTB* |
| Pteromyini | Petaurista alborufus | 0.198 | 0.370 | 0.076 | 0.074 | 0.426 | 0.135 | 0.120 | 0.214 | 0.202 | 0.230 | 0.248 | 0.241 | 0.117 |
|  | Petaurista hainana | 0.212 | 0.367 | 0.075 | 0.067 | 0.440 | 0.140 | 0.115 | 0.246 | 0.182 | 0.221 | 0.240 | 0.240 | 0.106 |
|  | Petaurista yunanensis | 0.194 | 0.339 | 0.075 | 0.079 | 0.559 | 0.128 | 0.114 | 0.218 | 0.193 | 0.227 | 0.238 | 0.235 | 0.107 |
|  | Pteromys volans | 0.175 | 0.339 | 0.081 | 0.084 | 0.450 | 0.137 | 0.119 | 0.202 | 0.201 | 0.231 | 0.247 | 0.242 | 0.096 |
|  | Trogopterus xanthipes | 0.207 | 0.370 | 0.089 | 0.095 | 0.459 | 0.147 | 0.130 | 0.280 | 0.255 | 0.231 | 0.255 | 0.298 | 0.113 |
|  | Hylopetes alboniger | 0.187 | 0.334 | 0.081 | 0.085 | 0.539 | 0.142 | 0.134 | 0.226 | 0.226 | 0.230 | 0.259 | 0.283 | 0.118 |
|  | Hylopetes phayrei | 0.187 | 0.367 | 0.090 | 0.099 | 0.427 | 0.147 | 0.136 | 0.237 | 0.244 | 0.230 | 0.252 | 0.288 | 0.118 |
|  | Priapomys leonardi | 0.192 | 0.367 | 0.087 | 0.085 | 0.441 | 0.154 | 0.128 | 0.212 | 0.229 | 0.231 | 0.255 | 0.245 | 0.122 |
|  | Glaucomys volans | 0.202 | 0.395 | 0.075 | 0.088 | 0.604 | 0.141 | 0.132 | 0.290 | 0.208 | 0.218 | 0.226 | 0.270 | 0.110 |
| Sciurini | Sciurus anomalus | 0.186 | 0.365 | 0.068 | 0.092 | 0.383 | 0.167 | 0.113 | 0.258 | 0.248 | 0.224 | 0.248 | 0.209 | 0.086 |
|  | Sciurus lis | 0.204 | 0.359 | 0.063 | 0.080 | 0.461 | 0.125 | 0.127 | 0.206 | 0.255 | 0.255 | 0.246 | 0.245 | 0.082 |
|  | Sciurus vulgaris | 0.195 | 0.348 | 0.067 | 0.083 | 0.441 | 0.131 | 0.124 | 0.205 | 0.250 | 0.263 | 0.240 | 0.256 | 0.082 |
|  | Hesperosciurus griseus | 0.198 | 0.395 | 0.068 | 0.086 | 0.413 | 0.156 | 0.120 | 0.248 | 0.283 | 0.214 | 0.243 | 0.265 | 0.103 |
|  | Sciurus aberti | 0.195 | 0.353 | 0.070 | 0.090 | 0.461 | 0.147 | 0.116 | 0.212 | 0.277 | 0.235 | 0.211 | 0.228 | 0.105 |
|  | Parasciurus arizonensis | 0.211 | 0.453 | 0.078 | 0.095 | 0.432 | 0.155 | 0.123 | 0.259 | 0.284 | 0.220 | 0.229 | 0.233 | 0.095 |
|  | Parasciurus nayaritensis | 0.212 | 0.356 | 0.079 | 0.095 | 0.437 | 0.154 | 0.126 | 0.254 | 0.293 | 0.225 | 0.222 | 0.231 | 0.096 |
|  | Sciurus carolinensis | 0.196 | 0.341 | 0.072 | 0.090 | 0.409 | 0.133 | 0.122 | 0.234 | 0.255 | 0.233 | 0.249 | 0.255 | 0.107 |
|  | Sciurus niger | 0.195 | 0.342 | 0.071 | 0.087 | 0.393 | 0.134 | 0.120 | 0.242 | 0.250 | 0.229 | 0.252 | 0.249 | 0.107 |
|  | Hadrosciurus ignitus | 0.182 | 0.340 | 0.067 | 0.073 | 0.399 | 0.150 | 0.110 | 0.207 | 0.297 | 0.210 | 0.242 | 0.221 | 0.102 |
|  | Hadrosciurus spadiceus | 0.191 | 0.349 | 0.072 | 0.083 | 0.422 | 0.143 | 0.128 | 0.226 | 0.292 | 0.211 | 0.230 | 0.219 | 0.105 |
|  | Hadrosciurus igniventris | 0.190 | 0.357 | 0.072 | 0.073 | 0.398 | 0.136 | 0.121 | 0.217 | 0.263 | 0.232 | 0.229 | 0.211 | 0.108 |
|  | Hadrosciurus pyrrhinus | 0.204 | 0.371 | 0.074 | 0.077 | 0.365 | 0.137 | 0.121 | 0.223 | 0.258 | 0.230 | 0.226 | 0.224 | 0.100 |
|  | Microsciurus flaviventer | 0.197 | 0.367 | 0.072 | 0.072 | 0.300 | 0.159 | 0.120 | 0.228 | 0.275 | 0.218 | 0.218 | 0.232 | 0.116 |
|  | Microsciurus sabanillae | 0.188 | 0.362 | 0.072 | 0.089 | 0.466 | 0.140 | 0.136 | 0.229 | 0.272 | 0.229 | 0.252 | 0.224 | 0.116 |
|  | Leptosciurus otinus | 0.189 | 0.378 | 0.073 | 0.090 | 0.428 | 0.142 | 0.123 | 0.230 | 0.275 | 0.244 | 0.235 | 0.272 | 0.101 |
|  | Leptosciurus similis | 0.203 | 0.373 | 0.078 | 0.092 | 0.380 | 0.142 | 0.107 | 0.241 | 0.273 | 0.225 | 0.228 | 0.253 | 0.100 |
|  | Leptosciurus mimulus | 0.178 | 0.358 | 0.076 | 0.084 | 0.417 | 0.141 | 0.147 | 0.227 | 0.265 | 0.214 | 0.247 | 0.251 | 0.105 |
|  | Leptosciurus pucheranii | 0.207 | 0.391 | 0.073 | 0.105 | 0.383 | 0.133 | 0.108 | 0.216 | 0.277 | 0.216 | 0.241 | 0.243 | 0.106 |
|  | Syntheosciurus brochus | 0.178 | 0.399 | 0.070 | 0.084 | 0.356 | 0.158 | 0.109 | 0.224 | 0.246 | 0.246 | 0.237 | 0.265 | 0.126 |
|  | Syntheosciurus granatensis | 0.181 | 0.366 | 0.068 | 0.081 | 0.358 | 0.154 | 0.117 | 0.257 | 0.243 | 0.230 | 0.241 | 0.260 | 0.104 |
|  | Echinosciurus variegatoides | 0.189 | 0.350 | 0.069 | 0.089 | 0.365 | 0.137 | 0.112 | 0.207 | 0.239 | 0.231 | 0.232 | 0.270 | 0.087 |
|  | Echinosciurus yucatanensis | 0.192 | 0.349 | 0.068 | 0.103 | 0.394 | 0.146 | 0.108 | 0.207 | 0.247 | 0.224 | 0.238 | 0.255 | 0.099 |
|  | Echinosciurus colliaei | 0.191 | 0.343 | 0.075 | 0.088 | 0.349 | 0.139 | 0.120 | 0.205 | 0.225 | 0.216 | 0.228 | 0.255 | 0.108 |
